# Retraction: Optimal performance selection of sustainable mobility service projects based on IFSS ‐ Prospect theory ‐ VIKOR: A case study of electric vehicle sharing program

**DOI:** 10.1371/journal.pone.0352738

**Published:** 2026-07-08

**Authors:** The PLOS One Editors

The *PLOS One* Editors retract this article [1] due to concerns about potential manipulation of the publication process. These concerns call into question the validity and provenance of the reported results. We regret that the issues were not identified prior to the article’s publication.

The Editors have additional concerns regarding the identity of the experts reported as surveyed in the Case study subsection of the Methodology section of [1], as well as the reproducibility of [1] based on the methodological details provided for the participant recruitment process.

Additionally, the articles cited as References 29, 31, 37, 39, 40, 45, and 66 were retracted after [1] was published.

All authors either did not respond directly or could not be reached.
